# GLUD1 inhibits hepatocellular carcinoma progression via ROS-mediated p38/JNK MAPK pathway activation and mitochondrial apoptosis

**DOI:** 10.1007/s12672-024-00860-1

**Published:** 2024-01-12

**Authors:** Qianwei Zhao, Mengdan Yu, Jinxia Li, Yaoyu Guo, Zexuan Wang, Kefei Hu, Fang Xu, Yixian Liu, Lili Li, Didi Wan, Ying Zhao, Jian Shang, Jintao Zhang

**Affiliations:** 1 Henan Institute of Medical and Pharmaceutical Sciences, Zhengzhou University, Zhengzhou, 450052 China; 2 School of Basic Medical Sciences, Academy of Medical Science, Zhengzhou University, Zhengzhou, 450052 China; 3 BGI College, Zhengzhou University, Zhengzhou, 450052 China; 4 Henan Key Medical Laboratory of Tumor Molecular Biomarkers, Zhengzhou University, Zhengzhou, 450052 China; 5 Henan Key Laboratory of Tumor Epidemiology and State Key Laboratory of Esophageal Cancer Prevention & Treatment, Zhengzhou University, Zhengzhou, 450052 China

**Keywords:** GLUD1, Metabolism, ROS, p38/JNK MAPK pathway, Apoptosis

## Abstract

Glutamate dehydrogenase 1 (GLUD1) is an important enzyme in glutamine metabolism. Previously, we found GLUD1 was down-regulated in tumor tissues of hepatocellular carcinoma (HCC) patients by proteomics study. To explore its role in the progression of HCC, the expressional level of GLUD1 was firstly examined and presented as that both the protein and mRNA levels were down-regulated in tumor tissues compared to the normal liver tissues. GLUD1 overexpression significantly inhibited HCC cells proliferation, migration, invasion and tumor growth both in vitro and in vivo, while GLUD1 knocking-down promoted HCC progression. Metabolomics study of GLUD1 overexpressing and control HCC cells showed that 129 differentially expressed metabolites were identified, which mainly included amino acids, bases, and phospholipids. Moreover, metabolites in mitochondrial oxidative phosphorylation system (OXPHOS) were differentially expressed in GLUD1 overexpressing cells. Mechanistic studies showed that GLUD1 overexpression enhanced mitochondrial respiration activity and reactive oxygen species (ROS) production. Excessive ROS lead to mitochondrial apoptosis that was characterized by increased expression levels of p53, Cytochrome C, Bax, Caspase 3 and decreased expression level of Bcl-2. Furthermore, we found that the p38/JNK MAPK pathway was activated in GLUD1 overexpressing cells. N-acetylcysteine (NAC) treatment eliminated cellular ROS and blocked p38/JNK MAPK pathway activation, as well as cell apoptosis induced by GLUD1 overexpression. Taken together, our findings suggest that GLUD1 inhibits HCC progression through regulating cellular metabolism and oxidative stress state, and provide that ROS generation and p38/JNK MAPK pathway activation as promising methods for HCC treatment.

**Supplementary Information:**

The online version contains supplementary material available at 10.1007/s12672-024-00860-1.

## Introduction

According to the 2020 Global Cancer Population Survey, liver cancer is the sixth most common cancer type and the third leading cause of cancer death [1]. Primary liver cancer includes hepatocellular carcinoma (HCC) (75–85%), intrahepatic cholangiocarcinoma (10–15%), and other rare types [2]. In recent years, the mortality rate of patients with liver cancer is still increasing at a rate of about 2–3% per year [3]. Usually, patients with liver cancer are diagnosed at an advanced stage and most of them have a poor prognosis. Therefore, it is urgently needed to develop highly sensitive and specific biomarkers and to explore the mechanism of key signaling pathways and factors in the development of liver cancer, which may bring new opportunities for the diagnosis, prognosis, and treatment of liver cancer.

Mitochondrial oxidative phosphorylation system (OXPHOS) deficiency and reactive oxygen species (ROS) production are the main causes of mitochondrial dysfunction [4], which is a research hotspot received extensive attention in recent years. Mitochondrial OXPHOS is the main source of cellular ATP that are generated during electron transfer process. ROS generated in mitochondria interact with cellular and mitochondrial components such as proteins, DNA, lipids, and other molecules, triggering mitochondrial dysfunction [5]. Mitochondrial dysfunction further leads to overproduction of ROS, which in turn induce oxidative stress and aggravate mitochondrial damage. ROS are closely related to various cancers, such as colorectal cancer, breast cancer, liver cancer, and cervical cancer [6–9]. It was found that ROS play dual roles in cancer initiation, progression, inhibition, and therapy. Low levels of ROS intend to stimulate cell proliferation and cancer progression, while excess ROS usually lead to cancer cell apoptosis [10].

Glutamate dehydrogenase 1 (GLUD1) is localized in the mitochondrial matrix, which is one of the key enzymes in glutamine metabolism process and uses NAD or NADP as a cofactor to catalyze the oxidative deamination of glutamate to *α*-ketoglutarate (*α*-KG) [11]. The changes of GLUD1 enzyme activity and its expression level are closely related to the occurrence and development of various diseases. The most reported case is hyperinsulinemic hypoglycemia (HH), which is caused by dominant heterozygous missense mutations in the *GLUD1* gene [12]. Besides, abnormal expression of GLUD1 affects the progression of breast cancer, gastric cancer, and prostate cancer [13–15]. As a metabolism-related enzyme, GLUD1 usually affects tumorigenesis and development by regulating cellular metabolic functions, which have been proved in glioblastoma, lung cancer, and renal clear cell carcinoma [16, 17].

In the previous study, we carried out quantitative proteomic detection of cancer and normal liver tissues of clinical HCC patients, and found that the protein expression level of GLUD1 in cancer tissues was significantly decreased [18]. It suggested that GLUD1 might function as a tumor suppressor during the progression of HCC. Earlier studies have shown that GLUD1 knockdown induces apoptosis of HCC cells in vitro, and GLUD1-mediated glutaminolysis is enhanced in HCC cells under glucose deprivation [19, 20]. However, the effect of GLUD1 on HCC progression and its molecular mechanism is still unclear. In this study, we confirmed down-regulation of GLUD1 in tumor samples from HCC patients. Besides, we found that GLUD1 overexpression enhanced mitochondrial OXPHOS activity and content of cellular ROS. Furthermore, excess ROS lead to activation of p38/JNK signaling pathway and induced mitochondrial apoptosis of HCC cells, which could be counteracted by ROS elimination with N-acetylcysteine (NAC) treatment.

## Materials and methods

### Human tissues

Paired (*n* = 24) tumor and adjacent noncancerous liver tissues were acquired from HCC patients in the First Affiliated Hospital of Zhengzhou University. This research obtained approval from the Research Ethics Committee of Zhengzhou University and consent of patients.

### Cell culture and lentivirus transduction

HCC cell lines including HepG2, Huh7, and SMMC-7721 were purchased from GeneChem Co. (Shanghai, China). SNU449 cell line was purchased from Guangzhou Cellcook Biotech Co. (Guangzhou, China). Cells were cultured in RPMI-1640 (HyClone, USA) or DMEM (Solarbio, China) medium (10% FBS) (Gibco, USA) at 37 °C and 5% CO_2_. Lentiviral particles of GLUD1 overexpressing or knockdown were purchased from GeneChem Co. (Shanghai, China) and infected into HCC cells. Stable cell lines were constructed by using puromycin (2 µg/mL). The sequences of GLUD1 shRNA and negative control are as follows: GLUD1 shRNA (sense: 5^,^-GCAGAGTTCCAAGACAGGATA-3^,^ ); Negative control (sense: 5^,^-TTCTCCGAACGTGTCACGT-3^,^ ).

### Protein extraction and Western blot

Cells were harvested and lysed in radio immunoprecipitation assay (RIPA) buffer (Solarbio, China) supplemented with phenylmethylsulfonyl fluoride (PMSF) (a serine protease inhibitor, Solarbio, China) for 30 min. BCA kit (Solarbio, China) was used to determine the concentration of protein in the supernatant. Proteins were separated on a 10% SDS-PAGE gel and transferred to a PVDF membrane (Merck Millipore, USA), and then incubated overnight with primary antibody (4 °C) after being blocked for 2 h in nonfat milk solution (5%). The membrane was reacted with TBST-diluted HRP-conjugated secondary antibody for 2 h at room temperature. Protein bands were detected by Amersham Imager 600 System (General Electric Company, USA) using ECL Substrate (Beyotime Biotech, China), and quantified with Image J software. The primary antibodies contain anti-GAPDH (BBI, China), anti-GLUD1 (BBI, China), anti-E-cadherin (1:1000, Abcam, USA), anti-Vimentin (1:1000, Boster, China), anti-Cytochrome C (ProteinTech, USA), anti-BCL2 (ProteinTech, USA), anti-BAX (Wanleibio, China), anti-Caspase 3 (Wanleibio, China), anti-JNK (Wanleibio, China), anti-phospho-JNK (Wanleibio, China), anti-p53 (Cell Signaling Technology, USA), anti-p38 (Cell Signaling Technology, USA) and anti-phospho-p38 (Cell Signaling Technology, USA).

### RNA extraction and quantitative reverse transcription PCR (RT-qPCR)

Cell total RNA was extracted using TRIzol reagent (Invitrogen, USA) following the manufacturer’s instructions and cDNA was synthesized by using the PrimeScript RT Reagent Kit (TaKaRa Clontech, China). The RT-qPCR was then performed by using the ChamQ^™^ Universal SYBR^®^ qPCR Master Mix (Vazyme Biotech, China) and QuantStudio 5 Real-Time PCR System (Thermo Scientific, USA). The mRNA expression levels were normalized to those of GAPDH and quantified by the comparative CT (2^−ΔΔCT^) method. The primers are synthesized by Shenggong (China) and sequences are provided as follows: *GLUD1* forward, 5^,^-GACGACCCCAACTTCTTCAAG-3^,^, reverse, 5^,^-TCCTCCACC AGCTTGTCCT-3^,^; Superoxide dismutase 1 (*SOD1)* forward, 5^,^-GATGACTTGGGCAAAGGTGGAAATG-3^,^, reverse, 5^,^-CCAATTACACCACAAGCCAAACGAC-3^,^; Superoxide dismutase 2 (*SOD2)* forward, 5^,^-CGCCCTGGAACCT CACATCAAC-3^,^, reverse, 5^,^-AACGCCTCCTGGTACTTCTCCTC-3^,^; Catalase (*CAT)* forward, 5^,^-CTCAGGTGCGGGCATTCTATGTG-3^,^, reverse, 5^,^-GGTGGACCTCAGTGAAGT TCTTGAC-3^,^; Glutathione peroxidase 1 (*GPX1)* forward, 5^,^-GCAACCAGTTTGGGCATCA GGAG-3^,^, reverse, 5^,^-CACCGTTCACCTCGCACTTCTC-3^,^; *GAPDH* forward, 5^,^-TCAAGAAGGTGGT GAAGCAGG-3^,^, reverse, 5^,^-TCAAAGGTGGAGGAGT GGGT − 3^,^.

### CCK8 proliferation assay

Cells (3000 cells/100 µL/well) were seeded in the 96-well plate. CCK-8 reagent (10 µL/well) was added to cell culture and then incubated for 2 h at 37 °C in the dark. The absorbance (A) value at 450 nm wavelength was detected with a microplate reader (Thermo, USA) every 24 h from 0 h to 96 h.

### Wound-healing assay

Cells (1 × 10^6^ cells/well) were seeded in the 6-well plate. When cell density reached 95%, three parallel lines were scratched onto the confluent cell layer and washed for two times with PBS. Cells were cultured in culture medium with 2% FBS. Images of migrating cells were sequentially taken every 24 h from 0 h to 48 h. The relative wound healing region among different sample groups was evaluated and compared.

### Cell migration and invasion assays

The migration assay was performed as follow: cells (5 × 10^4^ cells/well) were plated and cultured in the chambers with serum-free medium in the 24-well transwell plate (Corning, USA), while the culture medium in the well contained 20% FBS. After incubation for 24 h, cells were fixed for 20 min with 10% formalin. Then the chambers were washed with PBS and stained with crystal violet at room temperature for 20 min. Cell numbers in the chambers were analyzed with a microscope (magnification, x200; Olympus BX53, Japan). For invasion assay, Matrigel (BD Biosciences, USA) was diluted in serum-free medium (1:6) and added to the upper chamber before cells were seeded.

### Xenograft mice model

Animal experiments were performed according to the guidelines of the National Act on the Use of Laboratory Animals (P. R. China), and the procedures were approved by the Animal Ethics Committee of Zhengzhou University. 4 weeks old female nude mice were purchased from the Beijing Charles River Laboratory Technology Co. and upraised under SPF condition. Approximately 8 × 10^6^ SMMC-7721 or Huh7 cells were suspended in 100 µL PBS and inoculated subcutaneously into nude mice. Mice were sacrificed by cervical dislocation method 20 days after injection. Tumors in the mice were isolated, weighed, and photographed. Tumor volumes were evaluated with the formula: V = (width^2^ × length)/2. The tumor size did not exceed the permitted maximal volume of 1000 mm^3^.

### Immunohistochemistry (IHC) staining

Briefly, resected tumor tissue was dehydrated and embedded in paraffin. They were then thermally deparaffinized in EDTA buffer and blocked with 3% BSA. Tissue samples were incubated with anti-GLUD1 antibody overnight at 4 °C followed by secondary antibody at room temperature.

### Untargeted metabolomics

The cell samples were collected and grinded in tubes, then a mixture of methanol: water (4:1, v/v) solution (400 µL) was used to collect the metabolites. The mixture was first grinded for 6 min at −10 °C, 50 Hz, and then sonicated for 30 min 5 °C, 40 kHz. Later samples were centrifuged for 15 min at 4 °C, 13,000 g after incubation for 30 min at −20 °C. The quality control (QC) sample contains a mixture of 20 µL supernatant of each sample. The metabolites were analyzed using the UHPLC-Q Exactive HF-X system (Thermo, USA). It was repeated for 7 times with GLUD1 overexpressing and control SNU449 cells, respectively. The experimental process was carried out by Shanghai Majorbio Bio-pharm Technology Co.

### Detection of ROS content

Cells (1 × 10^5^ cells/well) were planted in the 12-well plate. 10 µM H_2_DCFDA or DHE probe was added to cells and cultured for 30 min at 37 °C in the dark. The fluorescence in cells was detected with Eclipse TS100 microscope (Nikon, Japan) and Image J software after being washed with PBS. For the detection of ROS content after NAC treatment, cells were incubated with NAC dilution (10 mM) for 36 h before DHE probe treatment.

### Oxygen consumption rate (OCR) measurement

Cells were seeded into a 96-well plate (8 × 10^4^/100 µL/well) and then cultured at 37 °C with 5% CO_2_ for overnight. Next day, the culture medium was discarded and cells were washed with PBS. Then the probe (BBoxiProbe^TM^ R01, BestBio, China) diluted with fresh medium and the oxygen barrier solution were sequentially added to the 96-well plate chamber at 37 °C, and the fluorescence intensity at the excitation wavelength of 468 nm was detected by the CLARIOstar Plus (BMG, Germany).

### Cell apoptosis rate detection

Cells were digested, collected and pelleted at 1200 rpm for 4 min, and then washed twice with cold PBS. Cell apoptosis rate was detected by Annexin V-APC/7-AAD apoptosis Detection Kit (KeyGEN; China). Briefly, cells were suspended with 100 µL binding buffer, and then 7-AAD and Annexin V-APC (5 µL) was added and cells were incubated at room temperature for 10 min in darkness. Later cell apoptosis rates were analyzed on ACEA NovoCyte3130 within 1 h.

### Statistical analysis

All statistical analyses were performed with GraphPad Prism 7.0. Data are shown as mean ± standard deviation (SD). Student^,^s t-test was used for the statistical analysis of different groups, and the *p* < 0.05 was considered as statistically significant.

## Results

### GLUD1 is down-regulated in tumor tissues of HCC patients

**Fig. 1:**
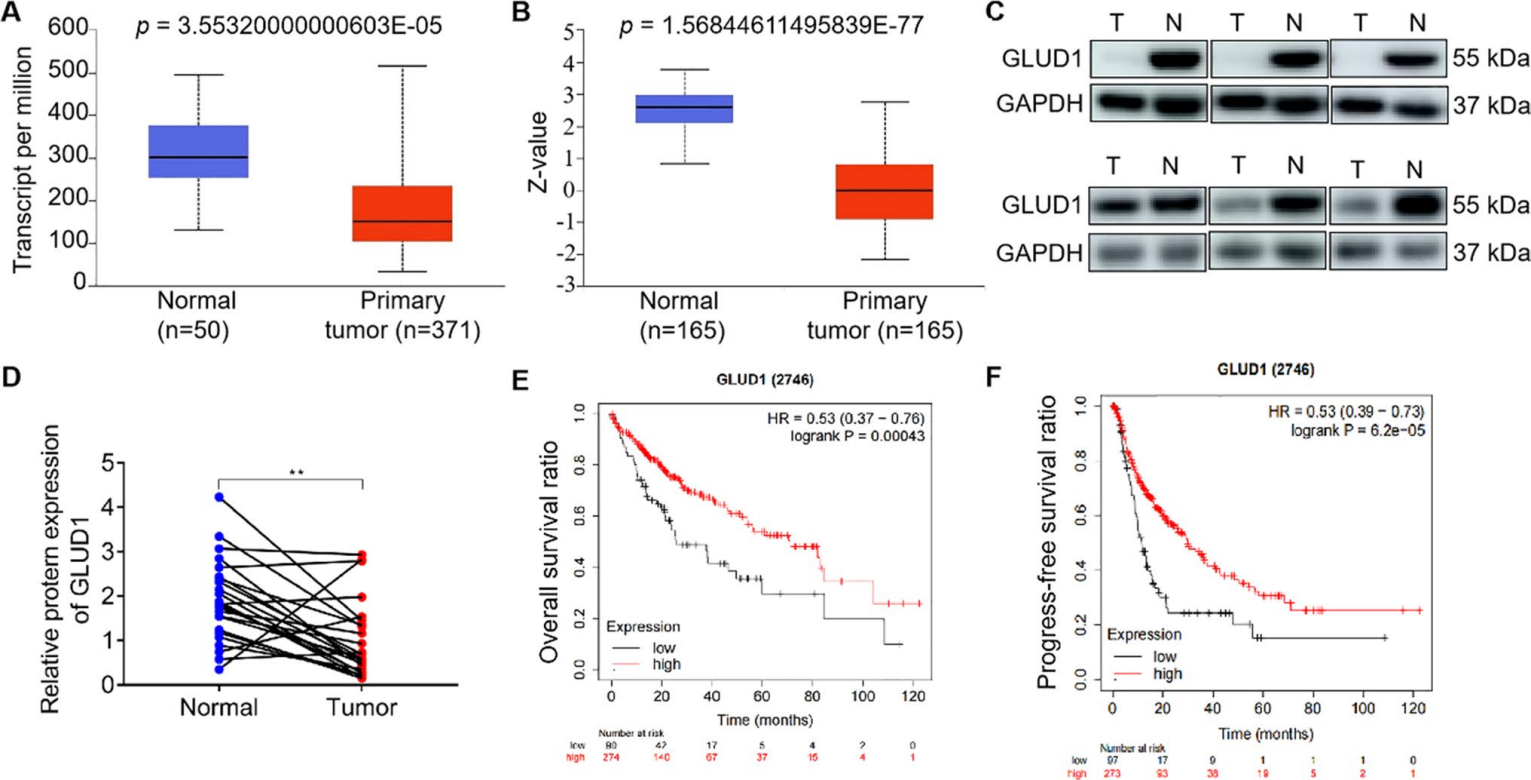
GLUD1 is down-regulated in HCC tumor tissues. **A** and **B** Analysis of GLUD1 transcription and protein levels based on the TCGA and CPTAC databases. **C** Detection of GLUD1 protein level in tumor and normal liver tissues by western blot. **D** Statistical analysis of GLUD1 relative expression level in paired samples from HCC patients (*n* = 24). **E** and **F** The relationship between GLUD1 expression level and OS or progression-free survival of HCC patients analyzed by the Kaplan-Meier survival curve. *TCGA* The Cancer Genome Atlas, *CPTAC* The Clinical Proteomic Tumor Analysis Consortium, *OS* Overall survivals. T represents tumor tissue, N represents normal liver tissue. ^**^p < 0.01

To explore the role of GLUD1 in regulating HCC progression, GLUD1 expression analysis was firstly performed with clinical HCC samples from the (The Cancer Genome Atlas) TCGA and (The Clinical Proteomic Tumor Analysis Consortium) CPTAC databases. As shown in Fig. 1A, B, both the mRNA and protein expression levels of GLUD1 were reduced in HCC tumor tissues compared to the normal liver tissues. Additionally, we collected another twenty-four pairs of tissue samples from HCC patients and detected the protein level of GLUD1. Results in Fig. 1C, D showed that compared to normal liver tissues, the protein level of GLUD1 was significantly reduced in tumor tissues. Moreover, higher expression level of *GLUD1* was associated with longer overall survivals (OS) and progression-free survivals of HCC patients based on Kaplan meier plotter database (Fig. 1E, F). Taken together, these results showed that GLUD1 was down-regulated in tumor tissues, and the high expression level of GLUD1 indicated good prognosis for HCC patients.

### GLUD1 overexpression inhibits tumorigenesis of HCC cells both in vitro and in vivo

**Fig. 2:**
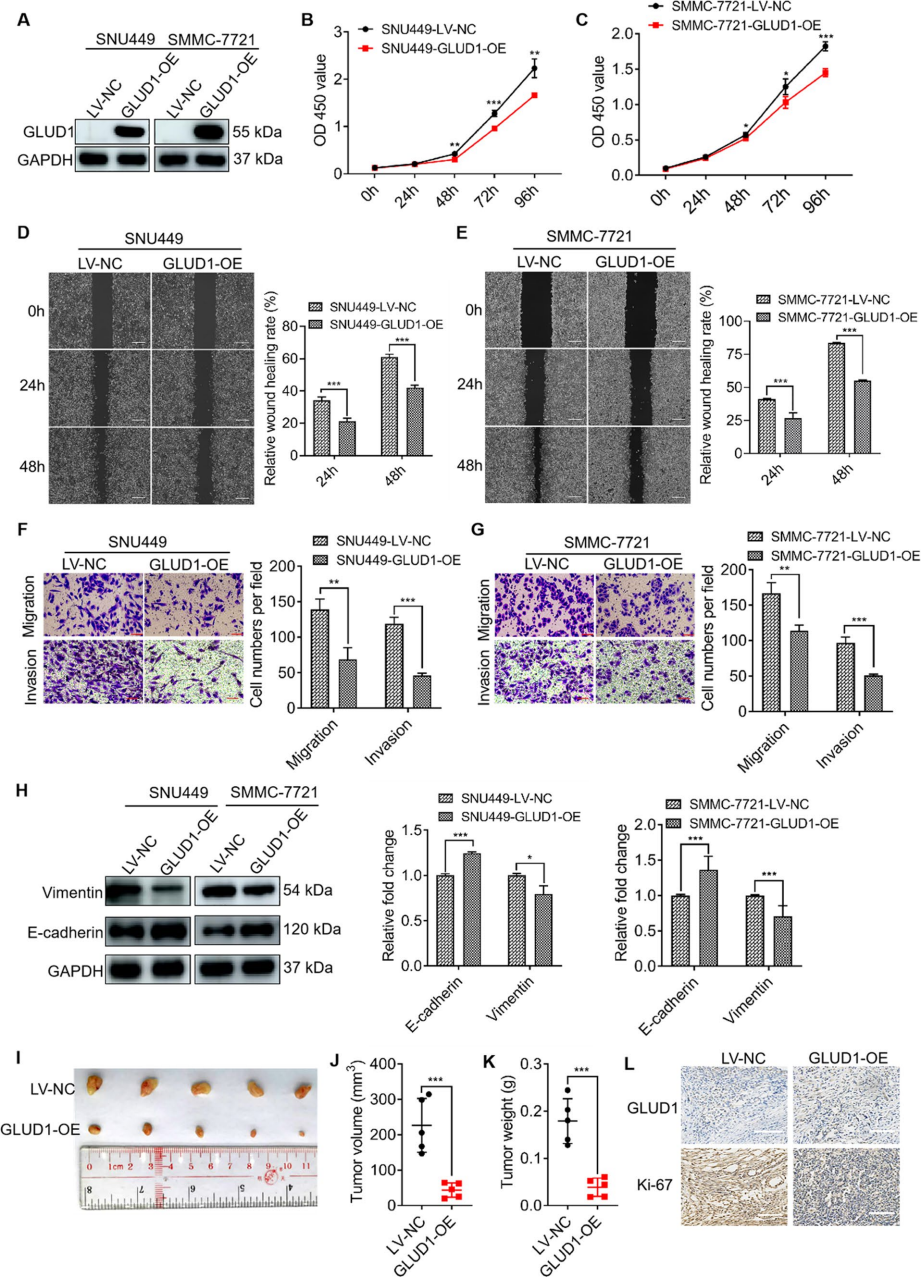
GLUD1 overexpression inhibits HCC cell proliferation, migration and invasion. **A** Western blot analysis of GLUD1 in GLUD1 overexpressing and control HCC cells. **B** and **C** CCK8 assay to detect the proliferation ability of GLUD1 overexpressing and control HCC cells. **D** and **E** wound healing (scale bars, 2 mm), **F** and **G** migration and invasion assays (scale bars, 400 μm) of GLUD1 overexpressing and control HCC cells. **H** Western blot analysis of E-cadherin and vimentin in GLUD1 overexpressing and control HCC cells. **I** Representative image, **J** and **K** Volume and weight of xenografts with GLUD1 overexpression or control HCC cell. **L** IHC staining of GLUD1 and Ki-67 in xenografts (scale bars, 100 μm). GLUD1-OE represents GLUD1 overexpressing HCC cell line, LV-NC represents control HCC cell line. ^*^p < 0.05, ^**^p < 0.01, ^***^p < 0.001

Next, we investigated the biological function of GLUD1 on HCC development both in vitro and in vivo. We detected the expression levels of GLUD1 in HCC cell lines including SNU449, SMMC-7721, Huh7, and HepG2. Results showed that the expression levels of GLUD1 in Huh7 and HepG2 cells were higher than those in SNU449 and SMMC-7721 cells (Fig. S1A). Thus, GLUD1 was overexpressed in SNU449 and SMMC-7721 cells by lentiviral transfection, which was confirmed with western blot and RT-qPCR analysis (Fig. 2A, Fig. S1B). CCK8 assay showed that GLUD1 overexpression significantly decreased the proliferation rate of HCC cells (Fig. 2B, C). Besides, GLUD1 overexpression inhibited wound closure, migration and invasion of HCC cells (Fig. 2D-G). E-cadherin and vimentin are two pivotal factors in regulating tumor cells migration and invasion. Results in Fig. 2H showed that E-cadherin was up-regulated while vimentin was down-regulated in GLUD1 overexpressing cells. Collectively, these results demonstrate that GLUD1 overexpression inhibits tumorigenesis of HCC cells in vitro.

Additionally, we investigated the influence of GLUD1 on HCC tumor growth in vivo. The xenograft mice model was generated by subcutaneous injection of SMMC-7721 cells overexpressing GLUD1 or control vectors into the flank of nude mice. About twenty days later, mice were sacrificed and the tumors were collected for analysis (Fig. 2I). Both the volume and weight of tumors with GLUD1 overexpression were significantly decreased, compared to those of the control group (Fig. 2J and K). Besides, IHC analysis showed that GLUD1 overexpression was maintained in the xenografts (Fig. 2L). Moreover, Ki-67 was down-regulated in xenografts with GLUD1 overexpression, which suggested the proliferation ability of HCC cells in vivo was decreased. In summary, these findings demonstrate that GLUD1 acts as an inhibitor in HCC progression.

### GLUD1 knockdown enhances HCC cell proliferation and metastasis abilities

**Fig. 3:**
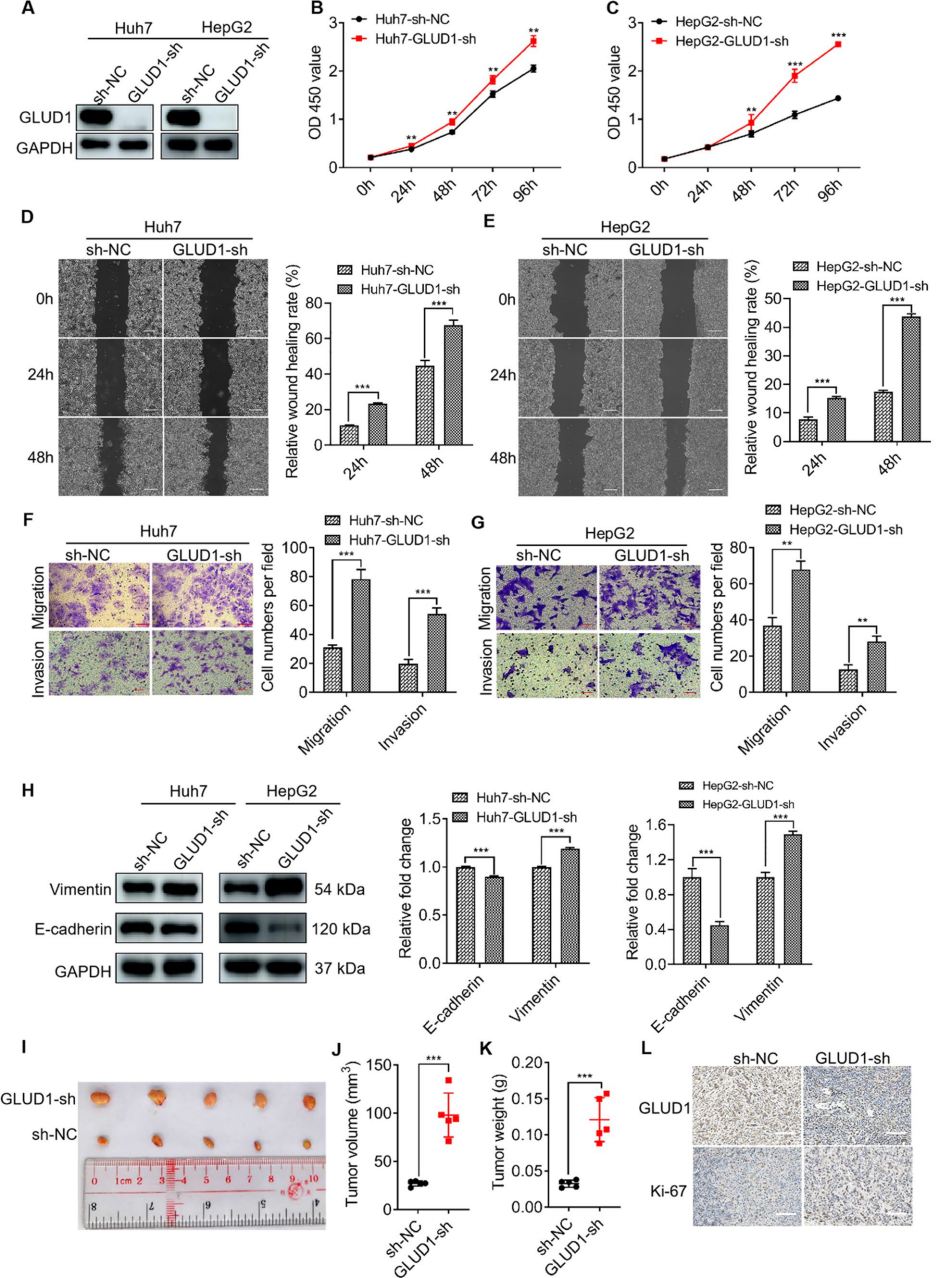
GLUD1 knockdown promotes HCC cell proliferation, migration and invasion. **A** Western blot analysis of GLUD1 in GLUD1 knockdown and control HCC cells. **B** and **C** CCK8 assay to detect the proliferation ability of GLUD1 knockdown and control HCC cells. **D** and **E** Wound healing (scale bars, 2 mm), **F** and **G** migration and invasion assays (scale bars, 400 μm) of GLUD1 knockdown and control HCC cells. **H** Western blot analysis of E-cadherin and vimentin in GLUD1 knockdown and control HCC cells. **I** Representative image, **J** and **K** Volume and weight of xenografts with GLUD1 knockdown or control HCC cells. **L** IHC staining of GLUD1 and Ki-67 in xenografts (scale bars, 100 μm). GLUD1-sh represents GLUD1 knockdown HCC cell line, sh-NC represents control HCC cell line. ^**^p < 0.01, ^***^p < 0.001

To further confirm the inhibitory function of GLUD1 in HCC progression, we constructed GLUD1 knocking-down and control cell lines with Huh7 and HepG2 cells by using lentiviral targeting GLUD1 (shRNA) and control vector. Knockdown of GLUD1 was confirmed by RT-qPCR and western blot analysis (Fig. 3A, Fig. S1C). Then we detected the cell viability using CCK8 and found that knockdown of GLUD1 significantly increased proliferation ability of cells (Fig. 3B, C). In addition, GLUD1 knockdown enhanced wound healing, migration and invasion abilities of HCC cells (Fig. 3D-G), which was consistent with the results that E-cadherin was down-regulated while vimentin was up-regulated in GLUD1 knockdown cells (Fig. 3H). These results suggest that knockdown of GLUD1 enhances the proliferation, mobility and invasion capabilities of HCC cells in vitro.

Next, we established the xenograft mice model and Huh7 cells with GLUD1 knockdown or control vector were implanted subcutaneously into nude mice. Twenty days later, mice were sacrificed and the tumors were collected for analysis (Fig. 3I). Results in Fig. 3J and K showed that the volumes and weights of xenografts from GLUD1 knockdown group were larger and higher than those of control group. Additionally, IHC analysis showed that GLUD1 knockdown was maintained in the xenografts where Ki-67 was overexpressed (Fig. 3L). Taken together, these results prove that GLUD1 is a tumor-suppressor and down-regulation of GLUD1 promoted HCC proliferation and metastasis both in vitro and in vivo.

### GLUD1 regulates the metabolism function of HCC cells

**Fig. 4:**
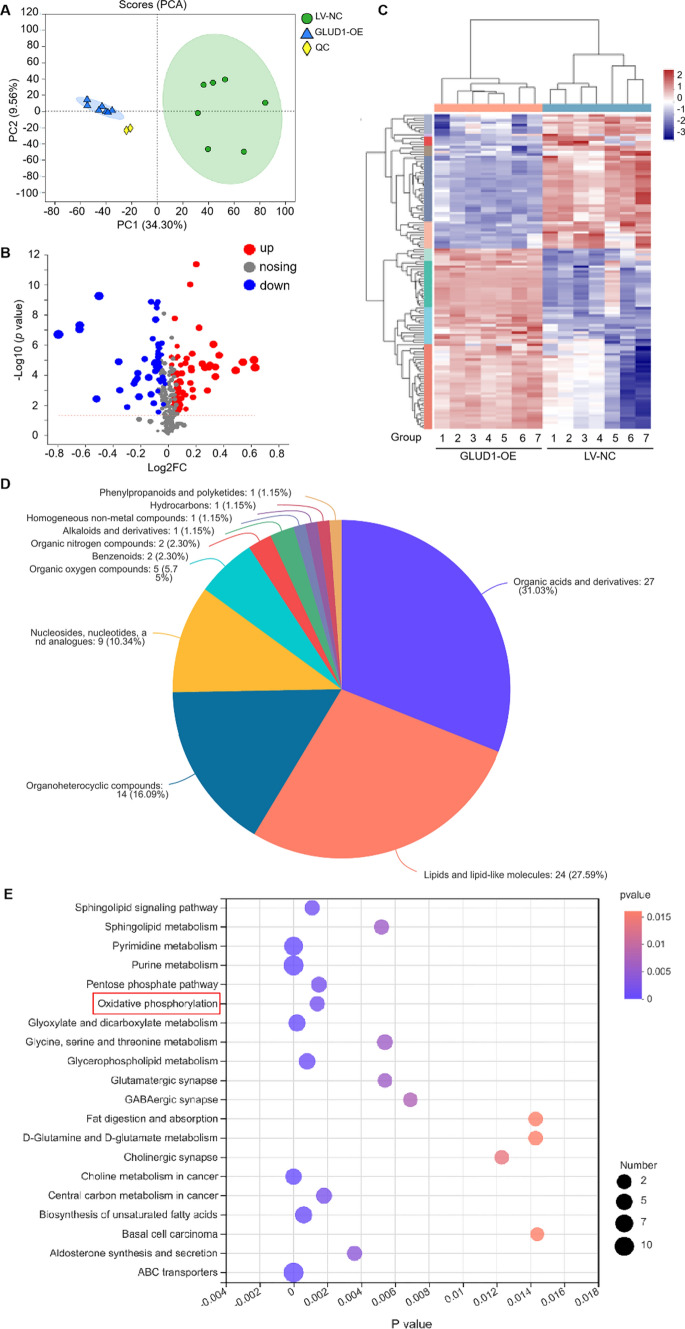
GLUD1 regulates cellular metabolism in HCC cells. **A** PCA of peak areas detected in positive-ion modes in QC, GLUD1 overexpressing SNU449 cells, and control SNU449 cells (*n* = 7). **B** Volcano plot of differentially expressed metabolites in GLUD1 overexpressing and control SNU449 cells (VIP ≥ 1, *p* < 0.05). **C** Heatmap of cluster analysis of differentially expressed metabolites. **D** Cluster analysis of differentially expressed metabolites by HMDB. **E** Enriched KEGG pathway (Top 20) of differentially expressed metabolites. *PCA* Principle component analysis, *QC* quality control, *HMDB* Human Metabolome Database, *KEGG* Kyoto Encyclopedia of Genes and Genomes. GLUD1-OE represents GLUD1 overexpressing HCC cell line, LV-NC represents control HCC cell line

GLUD1 is a key mitochondrial enzyme regulating glutamine metabolism. To further investigate the molecular mechanism of GLUD1 affecting HCC development, non-targeted metabolomics study was performed with GLUD1 overexpressing and control SNU449 cells. Notably, the result of Principle component analysis (PCA) showed clear separation between GLUD1 overexpressing and control cells, which indicated marked metabolic differences induced by GLUD1 overexpression (Fig. 4A, Fig. S2). Accordingly, metabolomics results showed that a total number of 408 metabolites were detected both in GLUD1 overexpression and control HCC cells. About 129 metabolites were identified with significant differences (VIP ≥ 1, *p* < 0.05), among which 49 metabolites were down-regulated and 80 metabolites were up-regulated in GLUD1 overexpressing HCC cells (Fig. 4B and C, Table S1). Based on the Human Metabolome Database (HMDB) analysis, the differentially expressed metabolites were mainly enriched into four clusters including organic acids and derivatives (31.03%), lipids and lipid-like molecules (27.59%), organoheterocyclic compounds (16.09%), nucleosides, nucleotides, and analogues (10.34%) (Fig. 4D). Moreover, Kyoto Encyclopedia of Genes and Genomes (KEGG) analysis of compounds classification showed that differentially expressed metabolites mainly included amino acids, bases, and phospholipids (Fig. S3). To better investigate the metabolic alterations in GLUD1 overexpressing cells, KEGG pathway enrichment analysis was performed. As shown in Fig. 4E, the differentially expressed metabolites were highly associated with purine metabolism, pyrimidine metabolism, ABC transporters, and another 17 metabolic pathways.

### GLUD1 enhances the OXPHOS function and aggravates oxidative stress in HCC cells

**Fig. 5:**
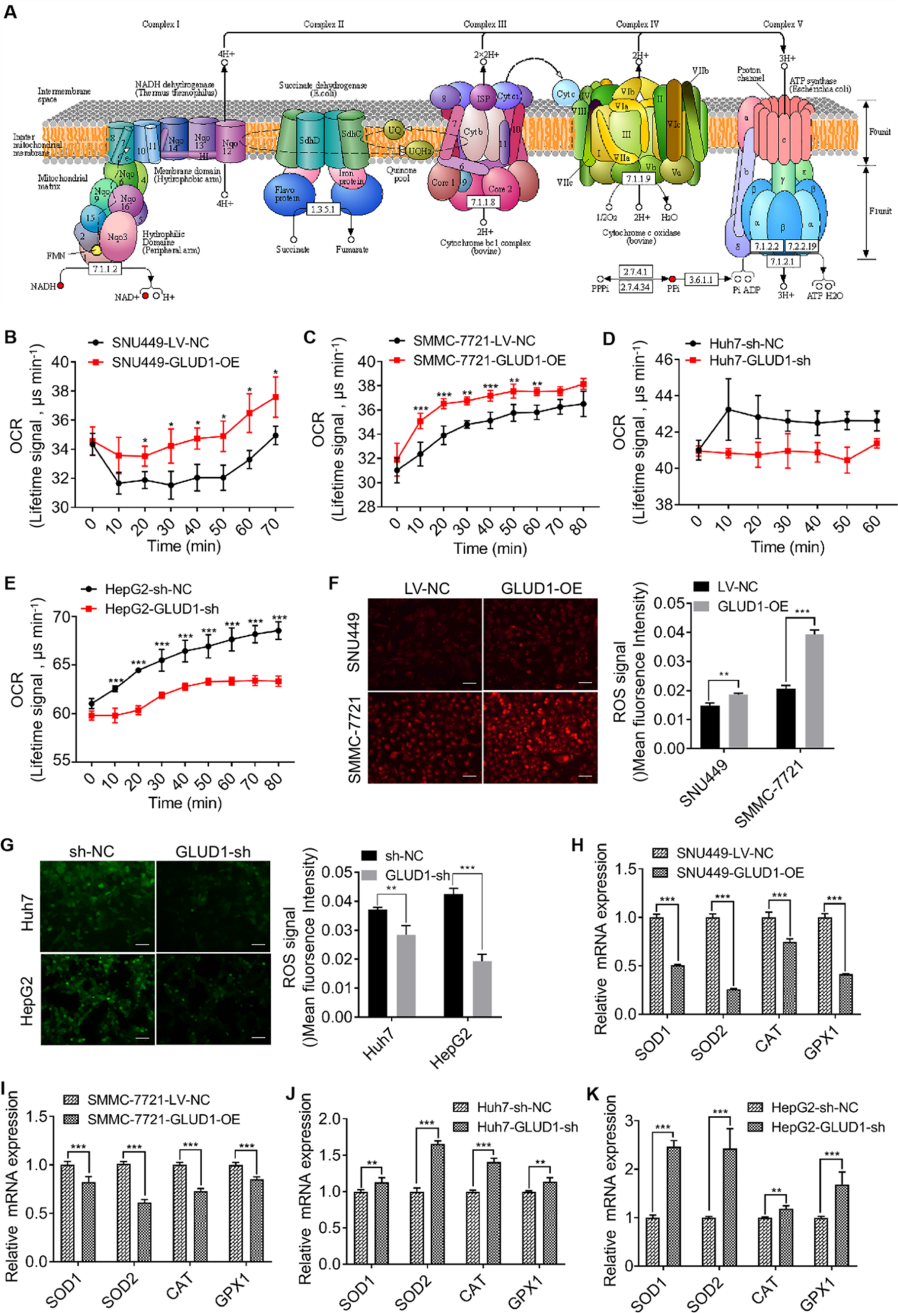
GLUD1 overexpression enhances OXPHOS activity and oxidative stress in HCC cells. **A** Schematic diagram of the OXPHOS process (substances marked in red represent the annotated differentially expressed metabolites). **B**, **C**, **D**, and **E** Detection of OCR in GLUD1 overexpression or knockdown HCC cells. **F** and **G** ROS content detection of HCC cells in GLUD1 overexpression (red fluorophores) or knockdown (green fluorophores) HCC cells. Scale bars, 200 μm. **H**, **I**, **J** and **K** The expression levels of antioxidant genes in GLUD1 overexpression or knockdown HCC cells were detected by RT-qPCR. GLUD1-OE represents GLUD1 overexpressing HCC cell line, LV-NC represents control HCC cell line, GLUD1-sh represents GLUD1 knockdown HCC cell line, sh-NC represents control HCC cell line. ^*^p < 0.05, ^**^p < 0.01, ^***^p < 0.001

Specially, we found that a certain content of differentially expressed metabolites were clustered into mitochondrial OXPHOS process. In Fig. 5A, the overall analysis of the OXPHOS pathway by KEGG website (https://www.kegg.jp) showed that the contents of NADH, NAD^+^ and PPi in GLUD1 overexpressing cells were significantly changed compared with the control cells. Besides, the differential metabolites involved in OXPHOS pathway were mainly produced by mitochondrial complexes I and V. Mitochondrial OXPHOS is an efficient metabolic process which provides most energy for cell growth [21]. To further verify the effect of GLUD1 on OXPHOS in HCC cells, we determined oxygen consumption by measurement of OCR. Results in Fig. 5B-E showed that the OCR was increased in GLUD1 overexpressing cells while decreased in GLUD1 knockdown cells. In summary, our findings demonstrate that GLUD1 has an effect on the metabolism of amino acid, fatty acid, and nucleoside, and enhances OXPHOS function of HCC cells.

Mitochondrial OXPHOS is the main source of ROS production, which induces oxidative stress and participates in cancer progression. To evaluate whether GLUD1 affects the state of oxidative stress, we detected cellular ROS content and found that GLUD1 overexpression increased ROS content while GLUD1 knockdown decreased ROS content in HCC cells (Fig. 5F, G). Moreover, the expression levels of antioxidants including *SOD1*, *SOD2*, *CAT*, and *GPX1* were measured by RT-qPCR. Results in Fig. 5H, I showed that the transcription levels of these antioxidants in GLUD1 overexpressing cells were decreased, which further confirmed increased oxidative stress with GLUD1 overexpression. Besides, we found that GLUD1 knockdown significantly increased the transcription levels of antioxidants, compared with the control cells (Fig. 5J, K). These findings demonstrated that GLUD1 plays an important role in regulation of oxidative stress by affecting ROS generation and antioxidants expression in HCC cells.

### GLUD1 induces mitochondrial apoptosis of HCC cells via ROS

**Fig. 6:**
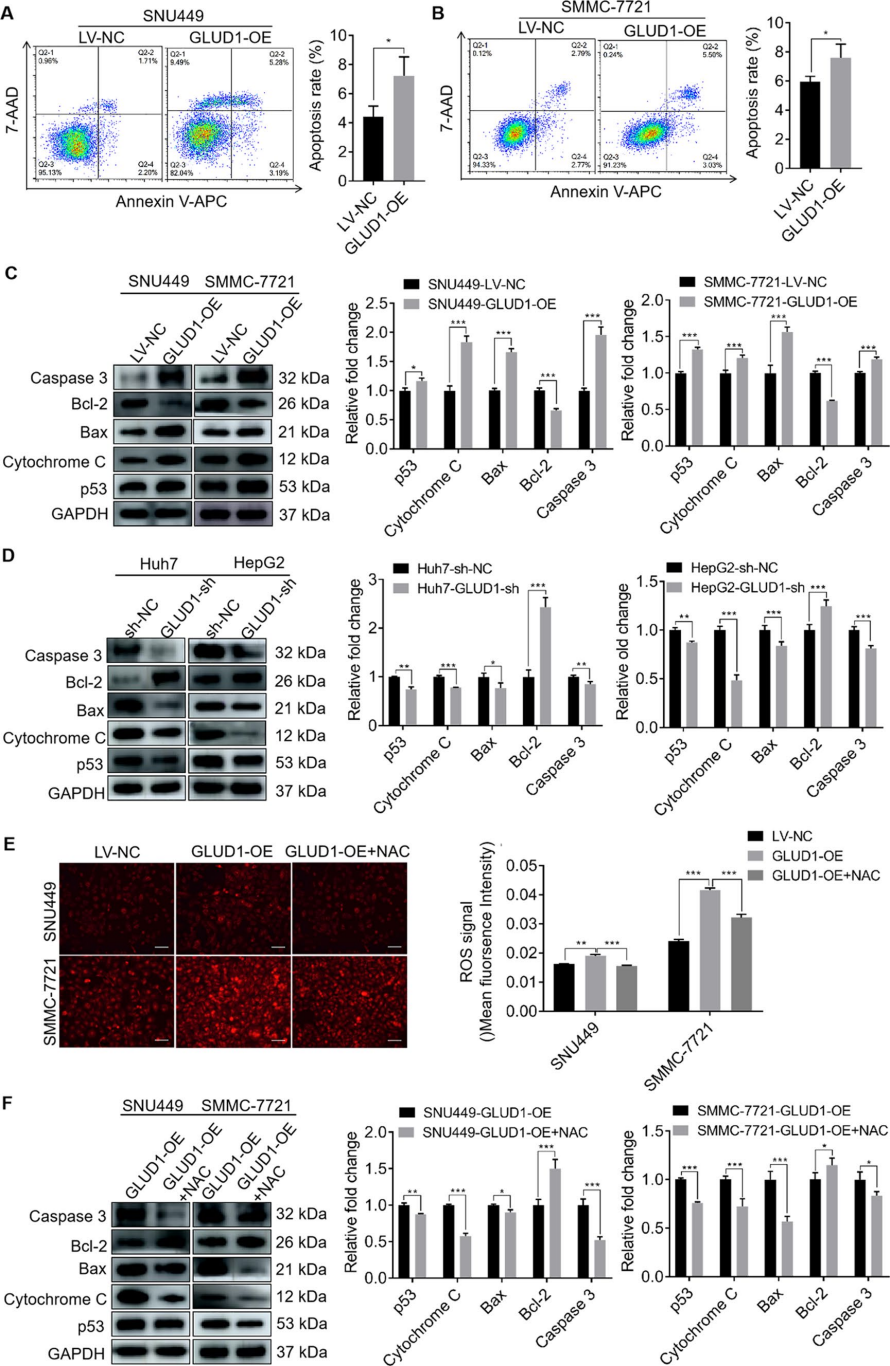
GLUD1 overexpression promotes HCC cells apoptosis via ROS. **A** and **B** Cell apoptosis rates detection of GLUD1 overexpressing and control cells. **C** and **D** Expression levels detection of p53, Cytochrome C, Bax, Caspase 3, and Bcl-2 by western blot in GLUD1 overexpression or knockdown HCC cells. **E** Detection of ROS content in GLUD1 overexpressing HCC cells after treatment with NAC. Scale bars, 200 μm. **F** Expression levels detection of p53, Cytochrome C, Bax, Caspase 3, and Bcl-2 by western blot in GLUD1 overexpressing HCC cells after treatment with NAC. GLUD1-OE represents GLUD1 overexpressing HCC cell line, LV-NC represents control HCC cell line, GLUD1-sh represents GLUD1 knockdown HCC cell line, sh-NC represents control HCC cell line. NAC: N-acetylcysteine. ^*^p < 0.05, ^**^p < 0.01, ^***^p < 0.001

 Studies have shown that oxidative stress caused by excessive ROS production in tumor cells promotes apoptosis through mitochondrial apoptosis pathway [22]. Then we detected the apoptosis ability of GLUD1 overexpressing and control HCC cells. Results in Fig. 6A and B showed that GLUD1 overexpression increased the apoptosis percent of HCC cells, compared with control cells. Furthermore, western blot was used to detect the expression levels of mitochondrial apoptosis-related proteins. The results in Fig. 6C presented that GLUD1 overexpression increased the expression levels of the pro-apoptotic proteins including p53, Cytochrome C, Bax and Caspase 3, and decreased the expression level of the anti-apoptotic protein Bcl-2. Besides, compared with the control cells, knockdown of GLUD1 in HCC cells significantly inhibited expression of pro-apoptotic proteins such as p53, Cytochrome C, Bax and Caspase 3, and promoted expression of anti-apoptotic protein Bcl-2 (Fig. 6D). Collectively, these results suggest that GLUD1 overexpression activates the mitochondrial apoptotic pathway in HCC cells.

To further confirm the function of ROS in inducing mitochondrial apoptosis of HCC cells, we treated GLUD1 overexpressing cells with NAC, which is a ROS scavenger, and examined its effect on mitochondrial apoptosis abilities of HCC cells. As shown in Fig. 6E, NAC treatment markedly decreased ROS content in GLUD1 overexpressing cells. In addition, we detected the expression levels of key factors involved in mitochondrial apoptosis process. Results in Fig. 6F showed that the expression levels of pro-apoptosis proteins including p53, Cytochrome C, Bax and Caspase 3 were decreased, while the expression level of anti-apoptosis protein Bcl-2 was up-regulated after NAC treatment in GLUD1 overexpressing cells. It indicates that mitochondrial apoptosis of HCC cells induced by GLUD1 overexpression was rescued by ROS elimination.

### ROS activates p38/JNK MAPK signaling pathway in GLUD1 overexpressing HCC cells

**Fig. 7:**
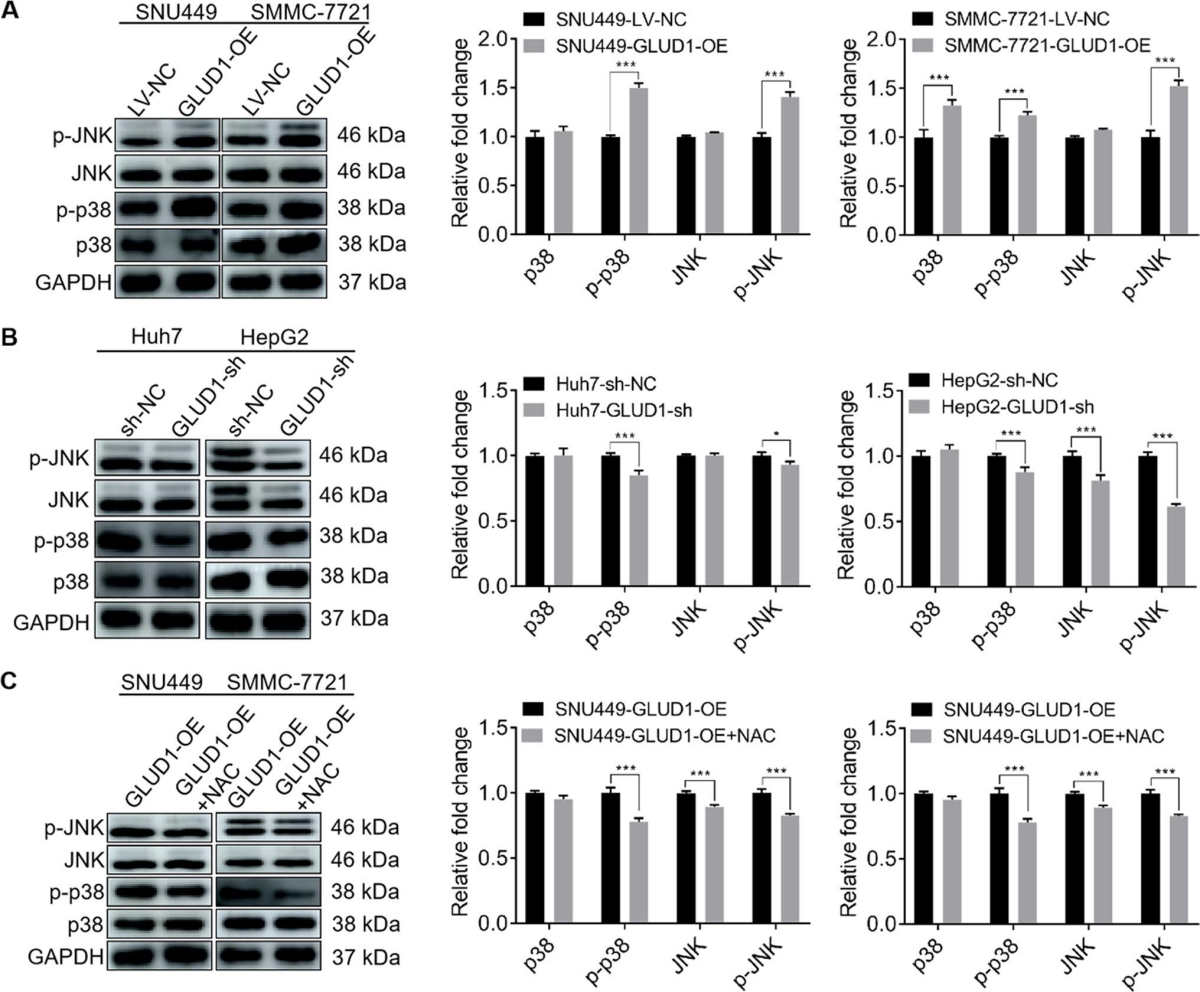
GLUD1 overexpression activates p38/JNK MAPK signaling pathway which is counteracted by NAC treatment. **A** and **B** Expression levels detection of p38, JNK, p-p38, and p-JNK by western blot in GLUD1 overexpression or knockdown HCC cells. **C** Expression levels detection of p38, JNK, p-p38, and p-JNK by western blot in GLUD1 overexpressing HCC cells after treatment with NAC. GLUD1-OE represents GLUD1 overexpressing HCC cell line, LV-NC represents control HCC cell line, GLUD1-sh represents GLUD1 knockdown HCC cell line, sh-NC represents control HCC cell line. NAC: N-acetylcysteine. ^*^p < 0.05, ^***^p < 0.001

 Earlier studies found that ROS-induced apoptosis was related to the activation of p38/JNK MAPK signaling pathway in tumor tissues including HCC [23, 24]. Then we detected the expression levels of p38/JNK MAPK signaling pathway-related proteins. Results in Fig. 7A and B showed that there were no significant differences in protein expression levels of p38 and JNK whether GLUD1 was overexpressed or knockdown. However, both the phosphorylation levels of p38 and JNK were increased in GLUD1 overexpressing cells, while GLUD1 knockdown inhibited the phosphorylation of p38 and JNK. These results demonstrate that overexpression of GLUD1 can activate the p38/JNK MAPK signaling pathway in HCC cells. Moreover, we detected the phosphorylation levels of p38 and JNK in GLUD1 overexpressing HCC cells after NAC treatment. Results in Fig. 7C showed that NAC treatment reduced the expression levels of phosphorylated p38 and JNK in GLUD1 overexpressing HCC cells. In conclusion, our findings demonstrate that GLUD1 overexpression promotes excess ROS generation and oxidative stress, which activates p38/JNK pathway and induces mitochondrial apoptosis of HCC cells.

## Discussion

GLUD1 functions in glutamine metabolism progress and provides *α*-KG for tricarboxylic acid (TCA) cycle, which is the central metabolic link which connects glucose, fatty acids, and amino acid metabolism [25]. Reports showed that dysregulation of both the expression level and activity of GLUD1 affected *α*-KG generation, and then regulated tumor cells proliferation and metastasis through affecting cellular metabolism [26]. Here we performed untargeted metabolomics study of GLUD1 overexpressing and control HCC cells and found that GLUD1 overexpression had an influence on cellular metabolism mainly including amino acid, fatty acid, and nucleoside metabolism pathways. These data suggest that GLUD1 participates in HCC progression through regulating cellular metabolism. Moreover, KEGG pathway enrichment showed that mitochondrial OXPHOS was one of the main metabolic pathways where differentially expressed metabolites were enriched in. Enhancement of OXPHOS capacity often leads to increased ROS content, which usually accompanies by decreased expression levels of antioxidant genes and aggravates the oxidative stress state of cancer tissues [27]. Here we found that GLUD1 overexpression not only enhanced mitochondrial OXPHOS function and ROS content, but also decreased the expression levels of antioxidant genes including *SOD1*, *SOD2*, *CAT,* and *GPX1* in HCC cells. These findings suggest that GLUD1 contributes to the cellular redox balance in HCC tissues, and GLUD1 overexpression aggravates oxidative stress state through enhancing OXPHOS activity and ROS content.

Usually, slightly higher levels of ROS act as signaling molecules and promote cancer development [28]. However, when excessive ROS are produced, they promote cell apoptosis and finally inhibit cancer cells proliferation and growth [29]. Here we found that Cytochrome C and Bax were up-regulated while Bcl-2 was down-regulated in GLUD1 overexpressing cells. The release of mitochondrial Cytochrome C is a critical step in mitochondrial apoptosis pathway as it is essential for the aggregation of adaptor molecule Apaf1 [30]. Besides, Bcl-2 family members including Bax form ion channels that allow the excretion of Cytochrome C, and induce sustained activation of caspase family proteins that are necessary for mitochondrial apoptosis [31]. Thus, our findings demonstrate that GLUD1 overexpression promotes mitochondrial apoptosis of HCC cells. It has been demonstrated in many kinds of cancers that inducing ROS production could induce cells apoptosis and prevent cancer cell proliferation and metastasis. Some ROS-promoting drugs showed inhibitory effect on HCC cells proliferation and tumor growth, which are expected to be candidates for clinical treatment of HCC, such as sorafenib, paclitaxel, curcumin, 5-fluorouracil, and so on [32–35]. Therefore, ROS content enhancement of cancer cells is an important and promising strategy for clinical treatment of HCC.

MAPKs signaling pathway consists of a serious of serine-threonine protein kinases, and participates in cell apoptosis and proliferation processes [36]. ERK, p38, and JNK are the main components of MAPKs signaling pathway, and it was reported that activation of MAPKs signaling pathway was closely related to mitochondrial apoptosis of HCC cells [37]. In this study, we found that GLUD1 overexpression promotes HCC cells apoptosis through ROS induced MAPKs signaling pathway activation. In recent years, studies on the activation of p38/JNK MAPK signaling pathway and the induction of tumor cell apoptosis have been confirmed and explored in liver cancer, lung cancer, pancreatic cancer, colorectal cancer, and so on [38–40]. MAPKs signaling pathway are taken as promising targets for cancer therapy, and some compounds that act as activators of MAPKs signaling pathway exhibit inhibitory effects on cancer progression through inducing mitochondrial apoptosis of cancer cells [41, 42]. Our findings verify the activation of MAPKs signaling pathway in inhibiting HCC progression and provide optional strategy for clinical HCC treatment. Besides, researches showed that MAPKs signaling pathway impacts the response of cancer cells to clinical therapy and is associated with drug resistance [43], which not only highlights the importance of MAPKs signaling pathway in cancer progression, but also indicates the influence of MAPKs signaling pathway on HCC treatment.

**Fig. 8:**
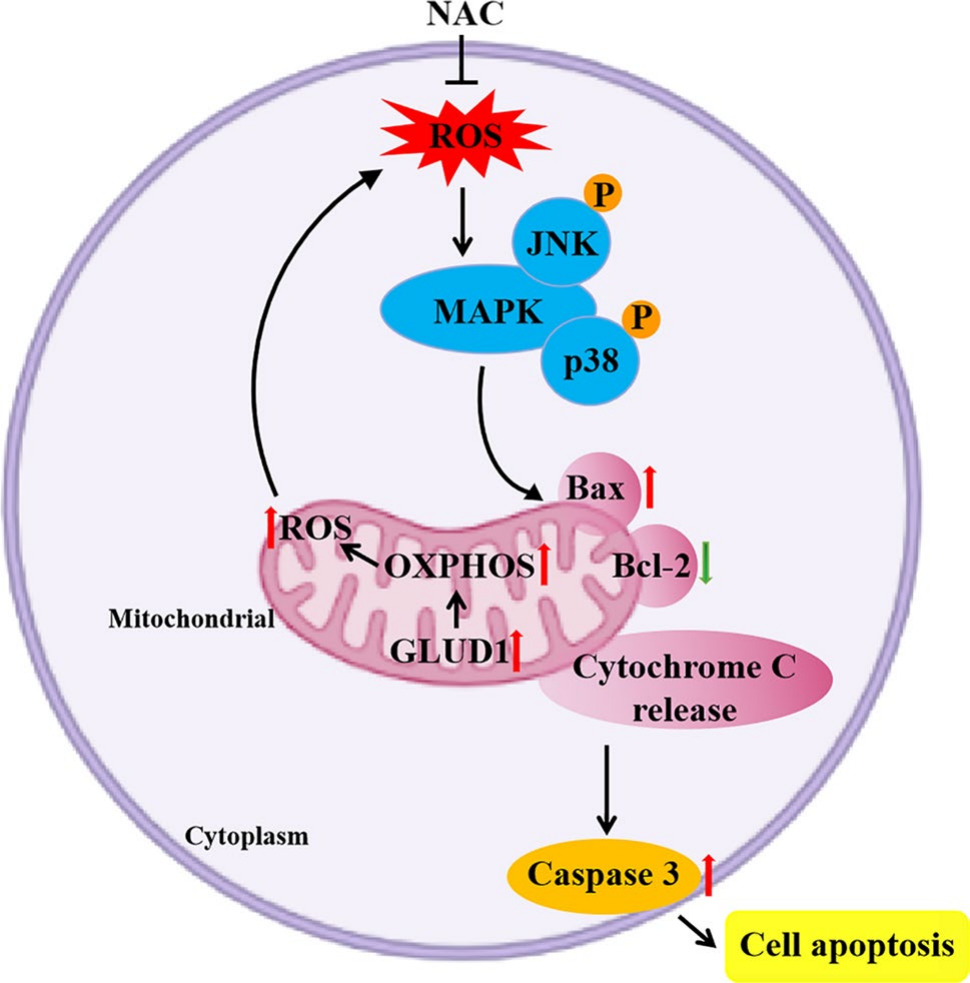
Schematic model of GLUD1 in promoting the p38/JNK MAPK signaling pathway activation and HCC cell apoptosis via enhanced mitochondrial OXPHOS capacity and ROS generation

 In conclusion, our study demonstrates that GLUD1 is down-regulated in tumor tissues of HCC patients and inhibits HCC progression both in vitro and in vivo. GLUD1 overexpression affects cellular metabolism and OXPHOS capacity which induces excess ROS generation and oxidative stress in HCC cells. Furthermore, p38/JNK MAPK signaling pathway is activated by ROS and induces mitochondrial apoptosis in GLUD1 overexpressing cells (Fig. 8). These findings provide GLUD1 as a promising biomarker for HCC prognosis, and suggest that ROS generation and MAPKs signaling pathway activation could be taken as candidate targets for HCC treatment.

### Supplementary Information

Supplementary material 1

Supplementary material 2

## Data Availability

The datasets generated and analyzed during the current study are available from the corresponding author on reasonable request.

## Abbreviations

GLUD1: Glutamate dehydrogenase 1
HCC: Hepatocellular carcinoma
TCA: Tricarboxylic acid
OXPHOS: Oxidative phosphorylation system
ROS: Reactive oxygen species
NAC: N-acetylcysteine
*α*-KG: *α*-ketoglutarate
HH: Hyperinsulinemic hypoglycemia
OS: Overall survivals
TCGA: The Cancer Genome Atlas
CPTAC: The Clinical Proteomic Tumor Analysis Consortium
RT-qPCR: Quantitative reverse transcription PCR
PCA: Principle component analysis
HMDB: Human Metabolome database
IHC: Immunohistochemistry
KEGG: Kyoto encyclopedia of genes and genomes
OCR: Oxygen consumption rate
SOD1: Superoxide dismutase 1
SOD2: Superoxide dismutase 2
CAT: Catalase
GPX1: Glutathione peroxidase 1
QC: Quality control

## References

[1] Sung H, Ferlay J, Siegel RL, Laversanne M, Soerjomataram I, Jemal A (2021). Global Cancer statistics 2020: GLOBOCAN estimates of incidence and mortality worldwide for 36 cancers in 185 countries. CA Cancer J Clin.

[2] Sia D, Villanueva A, Friedman SL, Llovet JM (2017). Liver cancer cell of origin, molecular class, and effects on patient prognosis. Gastroenterology.

[3] Wang W, Wei C (2020). Advances in the early diagnosis of hepatocellular carcinoma. Genes Dis.

[4] Lee HY, Nga HT, Tian J, Yi HS (2021). Mitochondrial metabolic signatures in hepatocellular carcinoma. Cells.

[5] Wu C, Zhang Z, Zhang W, Liu X (2022). Mitochondrial dysfunction and mitochondrial therapies in heart failure. Pharmacol Res.

[6] Jin F, Wu Z, Hu X, Zhang J, Gao Z, Han X (2019). The PI3K/Akt/GSK-3beta/ROS/eIF2B pathway promotes breast cancer growth and metastasis via suppression of NK cell cytotoxicity and tumor cell susceptibility. Cancer Biol Med.

[7] Wu Z, Zuo M, Zeng L, Cui K, Liu B, Yan C (2021). OMA1 reprograms metabolism under hypoxia to promote colorectal cancer development. EMBO Rep.

[8] Shao S, Duan W, Xu Q, Li X, Han L, Li W (2019). Curcumin suppresses hepatic stellate cell-induced hepatocarcinoma angiogenesis and invasion through downregulating CTGF. Oxid Med Cell Longev.

[9] Liu Y, Guo JZ, Liu Y, Wang K, Ding W, Wang H (2018). Nuclear lactate dehydrogenase A senses ROS to produce alpha-hydroxybutyrate for HPV-induced cervical tumor growth. Nat Commun.

[10] Bi L, Ren Y, Feng M, Meng P, Wang Q, Chen W (2021). HDAC11 regulates glycolysis through the LKB1/AMPK signaling pathway to maintain hepatocellular carcinoma stemness. Cancer Res.

[11] Nassar OM, Wong KY, Lynch GC, Smith TJ, Pettitt BM (2019). Allosteric discrimination at the NADH/ADP regulatory site of glutamate dehydrogenase. Protein Sci.

[12] Barrosse-Antle M, Su C, Chen P, Boodhansingh KE, Smith TJ, Stanley CA (2017). A severe case of hyperinsulinism due to hemizygous activating mutation of glutamate dehydrogenase. Pediatr Diabetes.

[13] Papadopoli D, Uchenunu O, Palia R, Chekkal N, Hulea L, Topisirovic I (2021). Perturbations of cancer cell metabolism by the antidiabetic drug canagliflozin. Neoplasia.

[14] Wu YJ, Hu ZL, Hu SD, Li YX, Xing XW, Yang Y (2019). Glutamate dehydrogenase inhibits tumor growth in gastric cancer through the notch signaling pathway. Cancer Biomark.

[15] He J, Mao Y, Huang W, Li M, Zhang H, Qing Y (2020). Methylcrotonoyl-CoA carboxylase 2 promotes proliferation, migration and invasion and inhibits apoptosis of prostate cancer cells through regulating GLUD1-P38 MAPK signaling pathway. Onco Targets Ther.

[16] Oizel K, Chauvin C, Oliver L, Gratas C, Geraldo F, Jarry U (2017). Efficient mitochondrial glutamine targeting prevails over glioblastoma metabolic plasticity. Clin Cancer Res.

[17] Shao J, Shi T, Yu H, Ding Y, Li L, Wang X (2021). Cytosolic GDH1 degradation restricts protein synthesis to sustain tumor cell survival following amino acid deprivation. EMBO J.

[18] Xu F, Jiang L, Zhao Q, Zhang Z, Liu Y, Yang S (2021). Whole-transcriptome and proteome analyses identify key differentially expressed mRNAs, miRNAs, lncRNAs and circRNAs associated with HCC. Oncogene.

[19] Zhou YJ, Yu HB, Cheng ST, Chen Y, He L, Ren JH (2022). Glutamate dehydrogenase 1 mediated glutaminolysis sustains HCC cells survival under glucose deprivation. J Cancer.

[20] Marsico M, Santarsiero A, Pappalardo I, Convertini P, Chiummiento L, Sardone A (2021). Mitochondria-mediated apoptosis of HCC cells triggered by Knockdown of Glutamate dehydrogenase 1: perspective for its inhibition through quercetin and permethylated anigopreissin A. Biomedicines.

[21] Ghosh P, Vidal C, Dey S, Zhang L (2020). Mitochondria targeting as an effective strategy for cancer therapy. Int J Mol Sci.

[22] Zhang B, Pan C, Feng C, Yan C, Yu Y, Chen Z (2022). Role of mitochondrial reactive oxygen species in homeostasis regulation. Redox Rep.

[23] Park S, Lim W, Bazer FW, Song G (2018). Naringenin suppresses growth of human placental choriocarcinoma via reactive oxygen species-mediated P38 and JNK MAPK pathways. Phytomedicine.

[24] Chang WT, Bow YD, Fu PJ, Li CY, Wu CY, Chang YH et al. A marine terpenoid, heteronemin, induces both the apoptosis and ferroptosis of hepatocellular carcinoma cells and involves the ROS and MAPK pathways. Oxid Med Cell Longev. 2021. 10.1155/2021/7689045 10.1155/2021/7689045 PMC7803406 33488943

[25] Plaitakis A, Kalef-Ezra E, Kotzamani D, Zaganas I, Spanaki C (2017). The glutamate dehydrogenase pathway and its roles in cell and tissue biology in health and disease. Biology (Basel).

[26] Jin L, Li D, Alesi GN, Fan J, Kang HB, Lu Z (2015). Glutamate dehydrogenase 1 signals through antioxidant glutathione peroxidase 1 to regulate redox homeostasis and tumor growth. Cancer Cell.

[27] Mailloux RJ (2020). An update on mitochondrial reactive oxygen species production. Antioxidants (Basel).

[28] Arfin S, Jha NK, Jha SK, Kesari KK, Ruokolainen J, Roychoudhury S (2021). Oxidative stress in cancer cell metabolism. Antioxidants (Basel).

[29] Zhu M, Jiang Y, Wu H, Shi W, Lu G, Cong D (2019). Gambogic acid shows anti-proliferative effects on non-small cell lung cancer (NSCLC) cells by activating reactive oxygen species (ROS)-induced endoplasmic reticulum (ER) stress-mediated apoptosis. Med Sci Monit.

[30] Antonsson B, Conti F, Ciavatta A, Montessuit S, Lewis S, Martinou I (1997). Inhibition of bax channel-forming activity by Bcl-2. Science.

[31] Liu L, Fu J, Li T, Cui R, Ling J, Yu X (2012). NG, a novel PABA/NO-based oleanolic acid derivative, induces human hepatoma cell apoptosis via a ROS/MAPK-dependent mitochondrial pathway. Eur J Pharmacol.

[32] Tu Y, Zhang W, Fan G, Zou C, Zhang J, Wu N (2023). Paclitaxel-loaded ROS-responsive nanoparticles for head and neck cancer therapy. Drug Deliv.

[33] Xu J, Ji L, Ruan Y, Wan Z, Lin Z, Xia S (2021). UBQLN1 mediates sorafenib resistance through regulating mitochondrial biogenesis and ROS homeostasis by targeting PGC1beta in hepatocellular carcinoma. Signal Transduct Target Ther.

[34] Wang L, Wang C, Tao Z, Zhao L, Zhu Z, Wu W (2019). Curcumin derivative WZ35 inhibits tumor cell growth via ROS-YAP-JNK signaling pathway in breast cancer. J Exp Clin Cancer Res.

[35] Li D, Song C, Zhang J, Zhao X (2022). ROS and iron homeostasis dependent ferroptosis play a vital role in 5-fluorouracil induced cardiotoxicity in vitro and in vivo. Toxicology.

[36] Luo Z, Zhu W, Guo Q, Luo W, Zhang J, Xu W (2016). Weaning Induced hepatic oxidative stress, apoptosis, and aminotransferases through MAPK signaling pathways in piglets. Oxid Med Cell Longev.

[37] Chang WT, Bow YD, Fu PJ, Li CY, Wu CY, Chang YH (2021). A marine terpenoid, heteronemin, induces both the apoptosis and ferroptosis of hepatocellular carcinoma cells and involves the ROS and MAPK pathways. Oxid Med Cell Longev.

[38] Chen X, Ma W, Yao Y, Zhang Q, Li J, Wu X (2021). Serum deprivation-response protein induces apoptosis in hepatocellular carcinoma through ASK1-JNK/p38 MAPK pathways. Cell Death Dis.

[39] Xiang Y, Ye W, Huang C, Lou B, Zhang J, Yu D (2017). Brusatol inhibits growth and induces apoptosis in pancreatic cancer cells via JNK/p38 MAPK/NF-kappab/Stat3/Bcl-2 signaling pathway. Biochem Biophys Res Commun.

[40] Kwak AW, Kim WK, Lee SO, Yoon G, Cho SS, Kim KT (2023). Licochalcone B induces ROS-dependent apoptosis in oxaliplatin-resistant colorectal cancer cells via p38/JNK MAPK signaling. Antioxidants (Basel).

[41] Jian KL, Zhang C, Shang ZC, Yang L, Kong LY (2017). Eucalrobusone C suppresses cell proliferation and induces ROS-dependent mitochondrial apoptosis via the p38 MAPK pathway in hepatocellular carcinoma cells. Phytomedicine.

[42] Wang JR, Luo YH, Piao XJ, Zhang Y, Feng YC, Li JQ (2019). Mechanisms underlying isoliquiritigenin-induced apoptosis and cell cycle arrest via ROS-mediated MAPK/STAT3/NF-kappaB pathways in human hepatocellular carcinoma cells. Drug Dev Res.

[43] Lee S, Rauch J, Kolch W (2020). Targeting MAPK signaling in cancer: mechanisms of drug resistance and sensitivity. Int J Mol Sci.

