# Supplementary material for: GLUD1 inhibits hepatocellular carcinoma progression via ROS-mediated p38/JNK MAPK pathway activation and mitochondrial apoptosis
Source: Discov Oncol. 2024 Jan 12;15:8. doi: 10.1007/s12672-024-00860-1 (PMC10786780; doi:10.1007/s12672-024-00860-1)
Supplement: Supplementary file 1 — Supplementary material 1 [file 12672_2024_860_MOESM1_ESM.docx]

**Additional Information**

**Figures and figure legends**

**
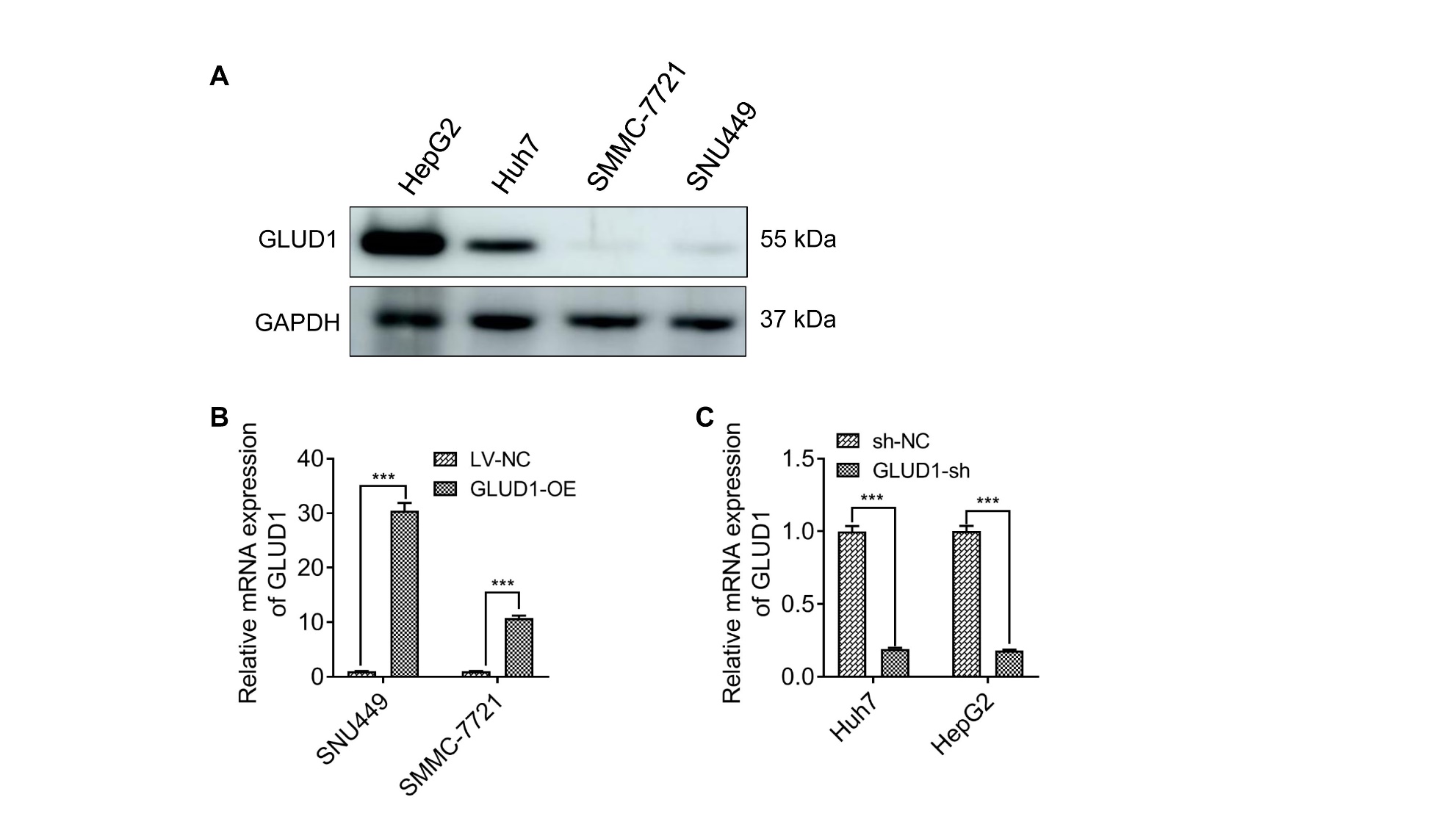
**

**Fig.S1 Detection of GLUD1 in HCC cell lines.** (A) Westwen blot analysis of GLUD1 in different HCC cell lines; (B) and (C) RT-qPCR analysis of GLUD1 in GLUD1 overexpressing (B) or knockdown (C) and control HCC cells. GLUD1-OE represents GLUD1 overexpressing HCC cell line, LV-NC represents control HCC cell line, GLUD1-sh represents GLUD1 knockdown HCC cell line, sh-NC represents control HCC cell line. ^***^*p* < 0.001.


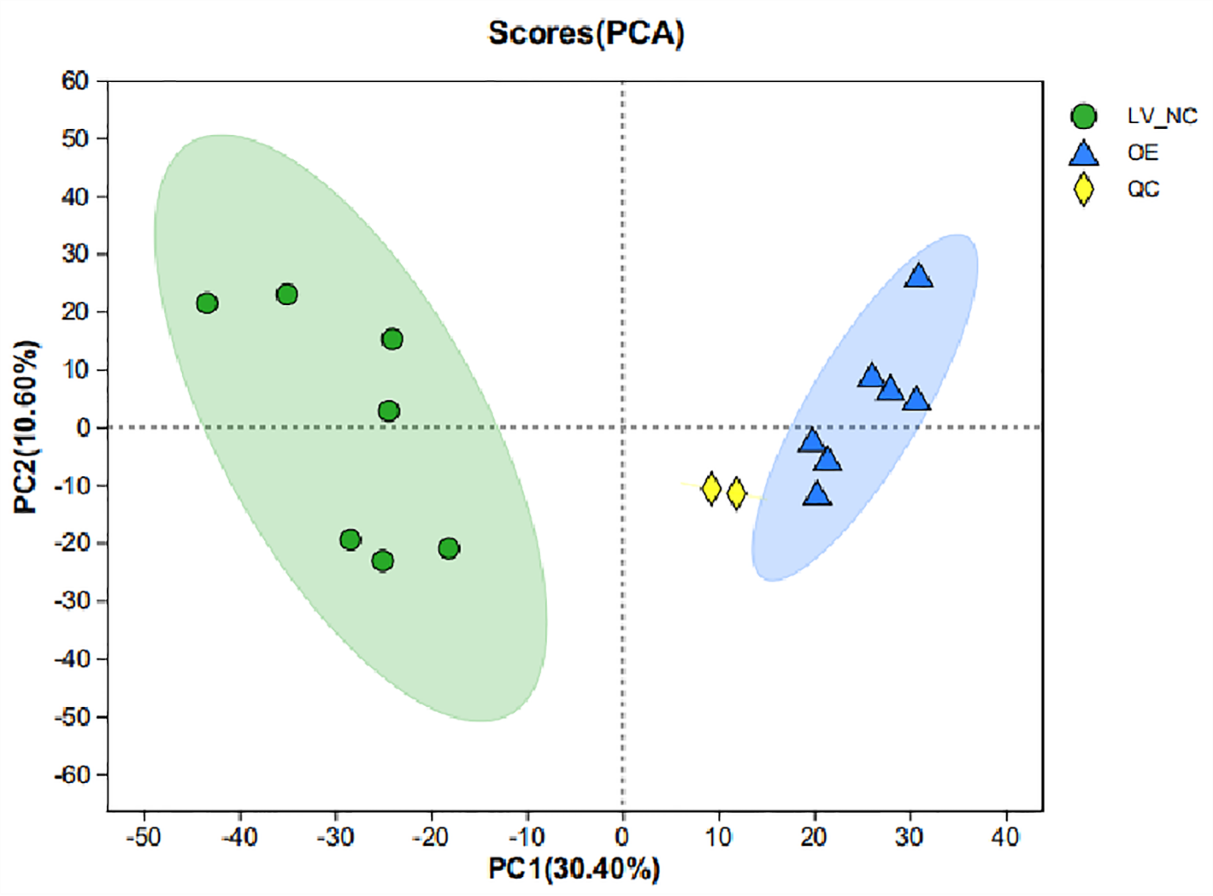


**Fig.S2 PCA of peak areas detected in negative-ion modes in QC, GLUD1 overexpressing SNU449 cells, and control SNU449 cells.**


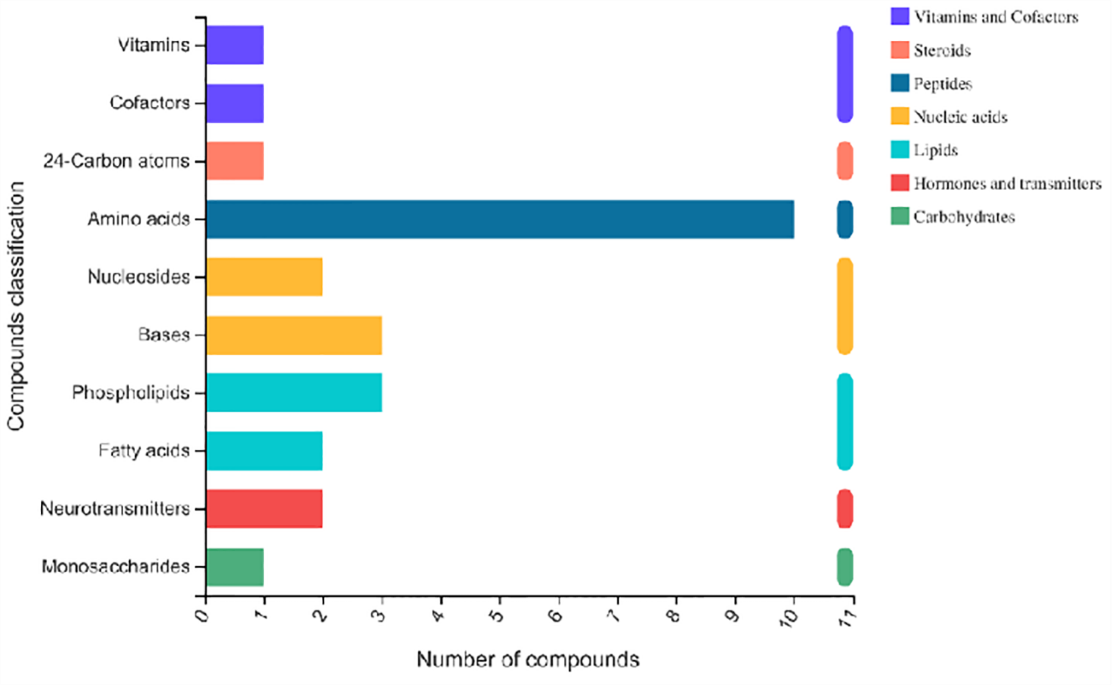


**Fig.S3 Compounds classification of differentially expressed metabolites.**
